# Spatial and Temporal Assessment of Pollen- and Seed-Mediated Gene Flow from Genetically Engineered Plum *Prunus domestica*

**DOI:** 10.1371/journal.pone.0075291

**Published:** 2013-10-01

**Authors:** Ralph Scorza, Alissa B. Kriss, Ann M. Callahan, Kevin Webb, Mark Demuth, Tim Gottwald

**Affiliations:** 1 U.S. Dept. of Agriculture, Appalachian Fruit Research Station, Kearneysville, West Virginia, United States of America; 2 U.S. Dept. of Agriculture, Agricultural Research Service, U.S. Horticultural Research Laboratory, Fort Pierce, Florida, United States of America; Virginia Tech, United States of America

## Abstract

Pollen flow from a 0.46 ha plot of genetically engineered (GE) *Prunus domestica* located in West Virginia, USA was evaluated from 2000–2010. Sentinel plum trees were planted at distances ranging from 132 to 854 m from the center of the GE orchard. Plots of mixed plum varieties and seedlings were located at 384, 484 and 998 m from the GE plot. Bee hives (*Apis mellifera*) were dispersed between the GE plum plot and the pollen flow monitoring sites. Pollen-mediated gene flow from out of the GE plum plot to non-GE plums under the study conditions was low, only occurring at all in 4 of 11 years and then in only 0.31% of the 12,116 seeds analyzed. When it occurred, gene flow, calculated as the number of GUS positive embryos/total embryos sampled, ranged from 0.215% at 132 m from the center of the GE plum plot (28 m from the nearest GE plum tree) to 0.033–0.017% at longer distances (384–998 m). Based on the percentage of GUS positive seeds per individual sampled tree the range was 0.4% to 12%. Within the GE field plot, gene flow ranged from 4.9 to 39%. Gene flow was related to distance and environmental conditions. A single year sample from a sentinel plot 132 m from the center of the GE plot accounted for 65% of the total 11-year gene flow. Spatial modeling indicated that gene flow dramatically decreased at distances over 400 m from the GE plot. Air temperature and rainfall were, respectively, positively and negatively correlated with gene flow, reflecting the effects of weather conditions on insect pollinator activity. Seed-mediated gene flow was not detected. These results support the feasibility of coexistence of GE and non-GE plum orchards.

## Introduction

Gene flow from genetically engineered (GE) crop plants to non-GE plants is a topic of interest and concern, and as such it has been the subject of a number of studies and review articles [1]–[14]. Concerns related to pollen-mediated transgene flow (PMTF) generally center around issues of developing weedy variants of wild species due to the acquisition of transgenes, or loss of species diversity due to increased fitness of GE hybrids [5], [10], [15], [4]. The majority of PMTF studies have been undertaken on agronomic crops such as soybean [8], maize [13], cotton [14], canola [15], and rice [16], or on herbaceous horticultural crops including tomato, sugar beet, strawberry [17], Chinese cabbage [10] and summer squash [11]. While there have been reviews [18] and theoretical and modeling studies [9], [19], there are few reports documenting PMTF in tree species with the exception of citrus [20], apple [21], and papaya, which is a relatively short-lived herbaceous tree species [22].

*Prunus* species (stone fruits) (family Rosaceae) constitute some of the world’s most important fruit and nut crops. Commercially produced species include almond (*P. dulcis*), apricot (*P. armeniaca*), sweet cherry (*P. avium*), sour cherry (*P. cerasus*), peach and nectarine (*P. persica*), and European and Japanese plum (*P. domestica and P. salicina*, respectively) with a total of 6.9 M ha under production worldwide, not including almonds. Plums are one of the most important stone fruits with 2.5 M ha under production worldwide with one species, *P. domestica*, accounting for over 95% of that acreage (http://faostat.fao.org, accessed Jan 22, 2013).

*P. domestica* is a woody perennial fruit tree grown in temperate zones of North and South America, Europe, and Asia. *P. domestica* is characterized by spring flowering and insect-mediated pollination. It is a hexaploid species and does not naturally cross-fertilize most other *Prunus* species [23]–[33] including apricot, almond, peach, sweet and sour cherry, or diploid plum (*P. salicina*) [34]. Cross-compatibility with the tetraploid species *P. spinosa* has been reported and the interspecific hybrid progeny display various levels of fertility. *P. domestica* is compatible with damson plums, which are classified as a variety or subspecies of *P. domestica* (http://plants.usda.gov/java/profile?symbol=PRDOI; http://www.plantnames.unimelb.edu.au/Sorting/Prunus_Pt2.html, accessed Feb 1, 2013).

Within *P. domestica*, genotypes display varying degrees of self- and cross-compatibility that are likely due to the interactions of *S*-alleles [35]. Bees of the species *Apis melifera* are reported to be the major pollination agent [36], [37] with bumblebees (*Bombus sp.*), other wild bees, and insects of the order Diptera, also providing pollination [38], [39].

Commercial varieties of plums and other stone fruits are generally developed through public, but in some cases private, breeding programs. Even the most successful new cultivars released from these breeding programs remain susceptible to a number of biotic (diseases and pests) and abiotic (temperature extremes, flooding, drought) constraints on production. Therefore, in addition to classical breeding for the development of improved varieties of stone fruits, genetic engineering (GE) technologies are currently being evaluated for genetic improvement of stone fruits. Research on the genetic engineering of resistance to *Plum pox virus* (PPV), one of the most serious diseases of stone fruits [40], has led to the development of a genetically engineered *P. domestica* plum cultivar that is highly resistant to PPV. This cultivar, ‘HoneySweet’, has received regulatory approval for cultivation in the U.S. [41] and is likely the forerunner of genetically engineered stone fruit cultivars to be developed in the near future by laboratories pursuing similar disease resistance strategies [42]–[44]. The release of new GE plum varieties brings to the forefront the question of transgene flow in *P. domestica*. An initial study submitted for regulatory consideration indicated that gene flow from a planting of GE plums was low (http://www.regulations.gov/#documentDetailD=APHIS-2006-0084-0004, accessed Nov 29, 2012). The current 11-year study, a continuation of that original study, was undertaken to: 1) provide a long-term, comprehensive view of pollen-mediated gene flow from *P. domestica* under typical field conditions; 2) provide observations on natural, seed-mediated gene flow; and 3) examine the spatial and temporal attributes of pollen-mediated gene flow under conditions of adequate pollinator densities.

## Materials and Methods

### GE *P. domestica* Planting (Plot T0)

**Figure 1:**
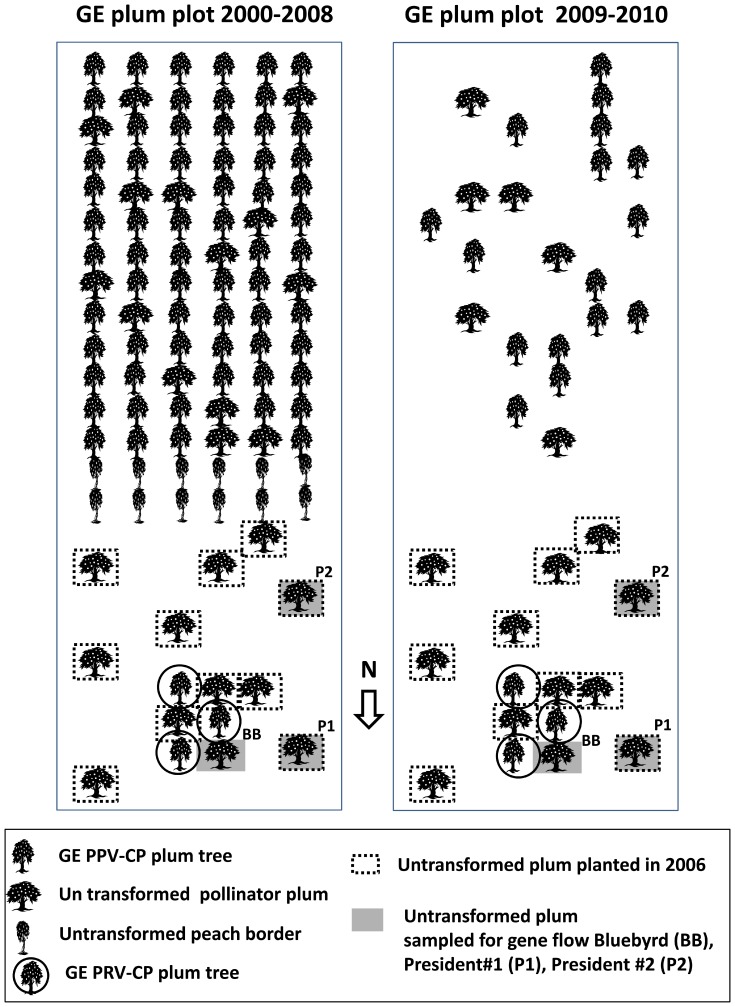
Plot T0 source block for gene flow from genetically engineered (GE) plum trees.

A 0.46 ha mixed planting of GE and non-GE plums (plot T0) was established on the grounds of the United States Department of Agriculture, Agricultural Research Service, Appalachian Fruit Research Station (USDA-ARS-AFRS) in Jefferson County West Virginia, latitude 39.352, longitude −77.884, elevation 172 m. Soils are classified as Hagerstown silt loam (fine, mixed, mesic Typic Hapludalf). The northern half of the plot was planted in 1992 under U.S. Animal and Plant Health Inspection Service (APHIS) permit 92-191-01 with trees containing the papaya ringspot virus (PRSV) coat protein (CP) gene, neomycin phosphotransferase (*NPT II*) and beta-D-glucuronidase (*uidA*, GUS) marker [45]. The southern half of the plot was planted in 1996 with trees containing the PPV CP, NPTII and GUS genes under APHIS permit 95-205-02 [46] (Fig. 1). In both subplots, non-GE plum trees were planted along with the GE trees to provide for cross-pollination. At the time of *P. domestica* planting, a double row of peach trees was planted around the perimeter of the plot. Peach trees were seedlings from open-pollination of ‘Bailey’ and were planted at 1.0–2.3 m between trees and 6.3 m between rows. Plum trees were spaced 2.3 m apart within the rows and rows were 6.3 m apart. Row direction was N-S. Trees were fertilized with 40 kg/ha nitrogen yearly in the first six years of growth with no fertilization thereafter. Chemical controls for insect pests and diseases were carried out when required under the recommendations of the West Virginia spray guide [47]. No insect control was applied during the flowering period. Pruning was minimal except for a more severe pruning to re-invigorate trees in the spring of 2009.

### Pollen-Mediated Gene Flow to Non-GE Plum Plots (S1, S2, S3, S4, S5, M1, M2, M3)

**Figure 2:**
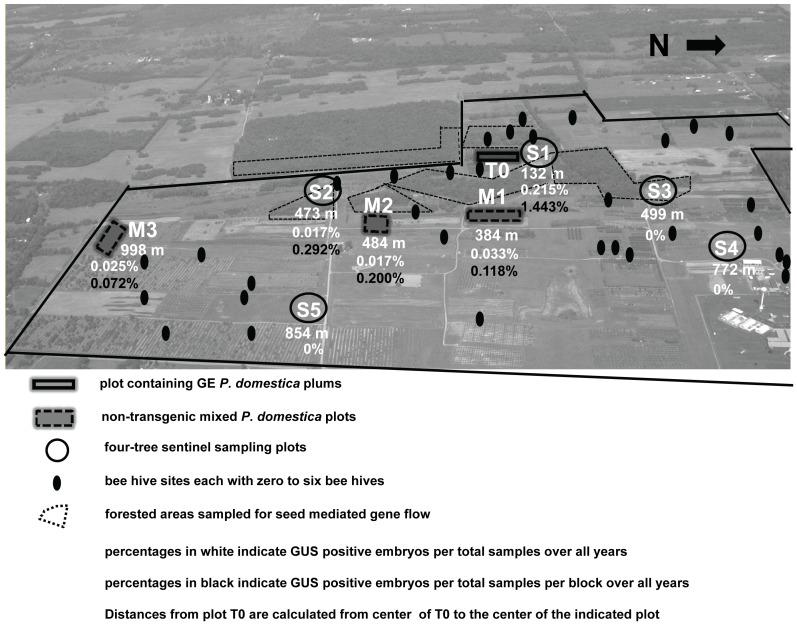
Experimental plantings of genetically engineered plums T0 and gene flow sampling plots (S1–S5; M1–M3). A total of 12,116 seeds were sampled for GUS assays over 11 years. Distances of sample plots from plot T0 and % GUS positive seeds/total seeds (white numbers) and % GUS positive seeds/total seeds/plot (black numbers ) are indicated. Borderline indicates USDA property line.

Over the 11 years of the study, gene flow was monitored in 52 trees. Sampled trees were located in nine plots in the orchard including those within the GE plum source block (T0) (Fig. 2). Individual trees were, on average, sampled in two of the 11 years which resulted in 115 samples during the course of the study.

*P. domestica* gene flow monitoring plots consisted of only *P. domestica* trees but were interspersed with plots of other tree fruit species including apple (*Malus* x *domestica*) and pear (*Pyrus communis*) and thus formed a discontinuous planting of *P. domestica*. Three of the plots (M1– M3) contained mixed genotypes of *P. domestica*. Plot M1 was a 1994 planting of 24 *P. domestica* seedlings, the products of mixed intraspecific hybridizations located 384 m E of the GE plot T0. Plot M2, planted in April 1979 was made up of seven trees of mixed varieties and selections 484 m SE of plot T0. Plot M3, 998 m SE of T0, consisted of 171 trees of mixed genotypes planted in May 1997 with ‘Seneca’ and ‘Italian Prune’ trees added to this plot in May 2003. Genotypes were selected for sampling based on a combination of traits including their proven compatibility or potential compatibility with ‘HoneySweet’ GE plum, time of bloom that would generally overlap a sequence of bloom dates of GE plums, and adaptation to the environment of the study site as indicated by consistent production. Cultivars included ‘Bluebyrd’, ‘Burja’, ‘Cacanska lepotica’, ‘Jojo’, ‘President’, four unique clones of ‘Pozegaca’, ‘Reine Claude’, ‘Sentibrskaja’, ‘Stanley’, and unnamed hybrid seedlings resulting from crosses of ‘Bluebyrd’ × ‘Reine Claude’, ‘Bluebyrd’ × ‘Stanley’, and ‘Ortenauer’ × ‘Stanley’. Of these, the ‘Pozegaca’ clones and ‘Burja’ were the latest blooming and only occasionally overlapped significantly in flowering time with the GE trees. Management of these monitoring plots was as described for plot T0.

Sentinel plots (S1, S2, S3, S4, S5) were planted in 2003 with two trees each of the cultivars ‘Seneca’ and ‘Italian Prune’. These varieties are self-incompatible and cross-incompatible with each other, but are sexually compatible with the GE variety ‘HoneySweet’. ‘HoneySweet’ was half-sibling to most other GE trees in plot T0 and it was therefore assumed that the compatibility status was similar for these trees. The inability of sentinel trees to self or cross pollinate with each other simplified sampling as fruit production in these plots could only occur as a result of gene flow from compatible *P. domestica* trees outside of the plot (GE or non-GE ). Monitoring of the sentinel plantings began in 2005 when they were fully flowering. Sentinel plots were at a distance of 132 m N (plot S1), 473 m SE (S2), 499 m NE (S3), 772 m NE (S4), and 854 m (SE) from the center of the GE plot T0 (Fig. 1).

### Assaying for Gene Flow

All GE trees in the GE plot expressed the beta-D-glucuronidase gene from *Escherichia coli* (*uidA*) [45], [46]. In order to sample pollen-mediated movement of the transgene, seeds were extracted from mature fruit of the trees being monitored in a particular year (6 trees in 2000, 3 in 2001, 11 in 2002, 13 in 2003, 16 in 2004, 7 in 2005, 21 in 2007, 18 in 2008, 13 in 2009, 8 in 2010). The number of seeds collected each year depended upon fruit load and resources to collect and process samples. Seeds were generally stored at 4°C for two weeks to one year prior to testing for *uidA* (GUS) expression. Tests on control seeds indicated that storage had no apparent effect on GUS expression (data not presented). Embryos were removed from the seeds and immersed in a solution consisting of 0.1 M sodium phosphate buffer (pH 7.0), 10 mM EDTA, 0.5 mM potassium ferricyanide, 0.1% Triton X-100, and 0.8 mM x-glucoronide. Following incubation at 37°C for one hour, embryos were visually assessed for GUS activity as evidenced by blue staining of the tissue [48]. Embryos derived from GE tree ‘HoneySweet’ were included in each assay as positive controls. Following immersion in this solution for up to 3 days, embryos that appeared positive for GUS activity were blotted on tissue paper to remove excess buffer, placed in 1.5 ml microfuge tubes and frozen in liquid nitrogen. Samples were then stored at −80°C until DNA was extracted.

#### DNA extraction

DNA from single embryos was extracted following the method of Kobayashi et al [49]. The embryo was ground directly in liquid nitrogen in the microfuge in the tube in which it had been frozen, using pestles designed to fit standard 1.5 ml tubes. (product # 749521-1590. Nalgene, Rochester, NY). One ml of extraction buffer was used and the extracted DNA was re-suspended in 20 µl water.

#### Gene detection

Real-Time PCR reactions to detect presence of specific genes were performed in triplicate utilizing 5 µl of TaqMan Universal PCR Master Mix (Invitrogen, Carlsbad, CA), 0.5 ul of the custom PrimeTime qPCR mix (IDT, Coralville, IA) and 1 µl of embryo DNA in a 10 µl reaction. The cycles were 2 min at 50°C, then 10 min at 95°C degrees followed by 45 cycles of 15 sec at 95°C and 1 min at 60°C on an ABI7900 sequence detector. The assay primers and ZEN Double-Quenched FAM probe in the Prime Time qPCR mix for each gene used are listed in Table 1. Dilution curves of leaf DNA from ‘Honeysweet’ (positive for CAB, PPV and ‘Honeysweet’), EF1 (positive for CAB and PRSV) and ‘Bluebyrd’ (positive for CAB) were run with each set of reactions. A dilution curve of pooled DNAs from ‘HoneySweet’ embryos and pooled DNAs from PRSV embryos was also run to verify the detectable amounts of DNA.

**Table 1:** Primer and probe combinations for analysis of pollen parent of transgenic embryos.

Name	Primer 1	Primer 2	Probe
Papaya ringspot virus coat protein (PRSV-CP)	TCTTCAGTACAATCCGCAACA	TCACTCCCTCATACCACTTCTC	56-FAM/TTTCTAACT/ZEN/CTCGTGCCACTCATTCACA/31ABkFQ
Plum pox virus (PPV)-CP	TTGGTATGAAGGAGTTAAGCGAG	TCCACTTGTGTTTCCCCATC	56-FAM/TGATGTCAC/ZEN/GGACGATGAAATGAGCA/31ABkFQ
HONEYSWEETPPV-CP hairpin transgene insert	GGTAGTTCCCACTGAATCAAAGGC	TTATTTCAACGCCAGTCCTGTCCC	56-FAM/TGGCAAGGA/ZEN/AATGTGCGAGTTCTGT/31ABkFQ
CAB Chlrophyll binding protein	TCAACTGGGCCCTTTCCAGTTACA	GGCGTTTGCTGAACTGAAGGTGAA	56-FAM/AATGGGCGG/ZEN/CTGGCAATGACTTCAAT/31ABkFQ

To determine the pollen parent for each of the individual GUS positive embryos a series of real-time PCR reactions was run to detect specific transgenes. All embryo DNAs were tested first with an assay for Chlorophyll AB binding protein to verify that the DNA was of high enough quality and to give a comparator for transgene DNA.

### Seed-mediated Transgene Flow

In the spring of 2012, 12.1 ha of native or naturalized forested areas on the USDA-ARS property were surveyed during the *P. domestica* bloom period for the presence of naturalized *P. domestica* trees. Volunteer plants had been allowed to naturally colonize these sites since 1979. The survey was based on observation of flowering *P. domestica* trees growing in the surveyed areas. An area south of the GE plot that bordered the USDA property was also visually surveyed but was not physically entered (Fig. 2). Three observations were made in the same year during the period of plum flowering.

### Pollinators

Due to unusually warm temperatures in the spring of 2012, *P. domestica* flowered prior to the installation of bee hives on the USDA property. This provided an opportunity to sample the natural pollinators of plum. Trees were sampled via sweep nets 10 times each on the first and second sampling dates (March 16 and 17) at a height of 2–2.5 m and four times each on the third date (March 30) to evaluate pollinators. On the first sampling date one GE tree in plot T0 was sampled, on the second date one GE tree in plot T0 and one non-GE tree in plot M3 were sampled, and on the third date two GE trees in plot T0 and three non-GE tree in plot M3 were sampled for a total of four trees sampled per category (GE and non-GE). Sweeps were conducted on sunny days with temperatures in the range of 24–27°C near the peak of *P. domestica* flowering when pollinators were abundant. Insects captured in sweep nets were counted and classified in most cases to genus and species.

### Honeybee Dispersal Effect on the Spatial Pattern of Gene Flow

Commercial bee hives of *A. melifera* were located on the USDA property for the cross-pollination of apple (*Malus x domestica*) and pear (*Pyrus communis*). The spatial pattern of the bees in the area encompassing the field plots evaluated in this study was investigated to determine the distribution of bees across the sampled trees based on GUS assays. Over the years surveyed, there were 32 sites within the grounds of the USDA-ARS-AFRS where bee hives were located, for a yearly total of between 37 and 70 bee hives. The number of bee hives at each site ranged from zero to six in a single year. However, the number of hives that were placed at a particular site was not recorded in all years. Using these data, a simulation (with 10,000 iterations) was conducted for each year to place the bee hives with unknown locations into sites. A log-normal probability function as described in Henry et al [50], with use of parameters from Capaldi et al. [51], was then used to describe the flight distances of bees from each of the bee hives to the sampled trees. The cumulative probability from all bee hives at distances that satisfy the set parameter values was determined for each sampled tree.

### Spatial Analysis of Gene Flow

The spatial pattern of gene flow was evaluated based on the distance each individual surveyed tree was from the GE plot (Plot T0). Gene flow was expressed as the proportion of GUS positive seeds per tree each year the tree was surveyed. However, gene flow was determined to be independent of year (data not shown) as there was no indication of a “build up” of positive seeds over time. Build up would not be expected because genes contributed via pollen are restricted to the embryo and not transmitted to the host tree. Therefore, all years (52 unique trees) were included in one model. The maximum proportion of positive seeds per tree over all of the years the tree was surveyed was used in the analysis to allow all results to be based on a maximum gene flow scenario. Several dispersal models were fit to the data with goodness of fit evaluated through residual plots and the coefficient of determination (R^2^).

### Weather Assessment during *P. domestica* Flowering

The USDA-ARS-AFRS manages a weather recording facility on the research station property. Data were recorded every 10 minutes daily and included air temperature (°C) rainfall (mm), relative humidity (%), wind speed (m/s), hourly evaporation (mm), and total photosynthetically active radiation (PAR) (µmol/m^2^/s). Two additional variables were calculated; the proportion of 10-minute increments during the bloom period each year where air temperature was greater than 9°C, and the proportion of 10-minute increments during the bloom period each year when it was not raining and air temperature was greater than 9°C. These additional weather variables were investigated since precipitation and temperature have been reported to be related to honeybee activity [52]–[54]. Climatological data were compiled for 7 years (2004–2010) during a 1 to 2 week period of *P. domestica* bloom, which varied between years (data not presented). Pearson correlation coefficients were utilized to investigate the inter-annual association between each of the weather variables and the proportion of trees that were found to have GUS positive seeds and the average and maximum dispersal distance. The correlations were weighted based on the number of sampled trees each year. For each weather variable, means for both years with recorded gene flow out from plot T0 (2006, 2007, 2008) and without gene flow (2004, 2005, 2009, 2010) were determined.

## Results

### Transgene Flow within the GE Planting T0

In 2005 and 2010, 912 embryos were sampled from non-GE pollinator trees within the GE plot. In 2005, a ‘Bluebyrd’ tree nearest several GE trees produced 39% GUS positive embryos (195 of 500 seeds) (Fig. 1). In 2010, 4.9% of the seeds collected from the same ‘Bluebyrd’ tree were GUS positive (8 of 164). The ‘President’ trees samples in 2010 consisted of one sample of 48 seeds from President tree #1, 97 seeds from President tree #2 and a sample of 103 seeds that were a mixed collection from trees #1 and #2 of approximately equal quantities of seed from each tree. ‘President’ tree #1 produced 6.3%, ‘President’ tree #2 produced 9.3% and the bulk sample from ‘President’ #1 and #2 produced 3.9% GUS positives (Table 2). Of 218 GUS positive embryos analyzed by PCR from plot T0, 71% tested positive for receiving pollen from PRSV-CP trees, 26% from PPV-CP trees, 1% from ‘HoneySweet’, and <2% from the vector control.

**Table 2:** Gene flow based on sampled trees within plots for the mixed variety plots (M1–M3), and the sentinel plots (S1–S5) from 2000–2010.

Plot	Range of distance from sampled trees to GE plot (m)	Number of unique trees sampled	Total number of trees sampled	Number of sampled trees with GUS positive embryos	Total Number of embryos sampled	Total Number of GUS Positive embryos	Range of proportion of GUS positive embryos per sampled tree
M1	384.3–384.8	20	52	4	3385	4	0–0.022
M2	478.2–490.0	3	7	1	918	2	0–0.004
M3	975.7–1032.8	18	29	1	4192	3	0–0.012
S1	131.8–132.3	3	8	4	1802	26	0–0.122
S2	468.5–479.5	3	7	2	686	2	0–0.012
S3	495.6–501.7	2	5	0	691	0	0
S4	768.7–774.4	2	6	0	440	0	0
S5	853.6	1	1	0	21/	0	0

1/ Low seed number was due to the absence of self and cross compatibility of sentinel trees and lack of gene flow from compatible plums outside of the S5 plot (GE or non-GE).

### Gene Flow Out from the GE Planting

**Figure 3:**
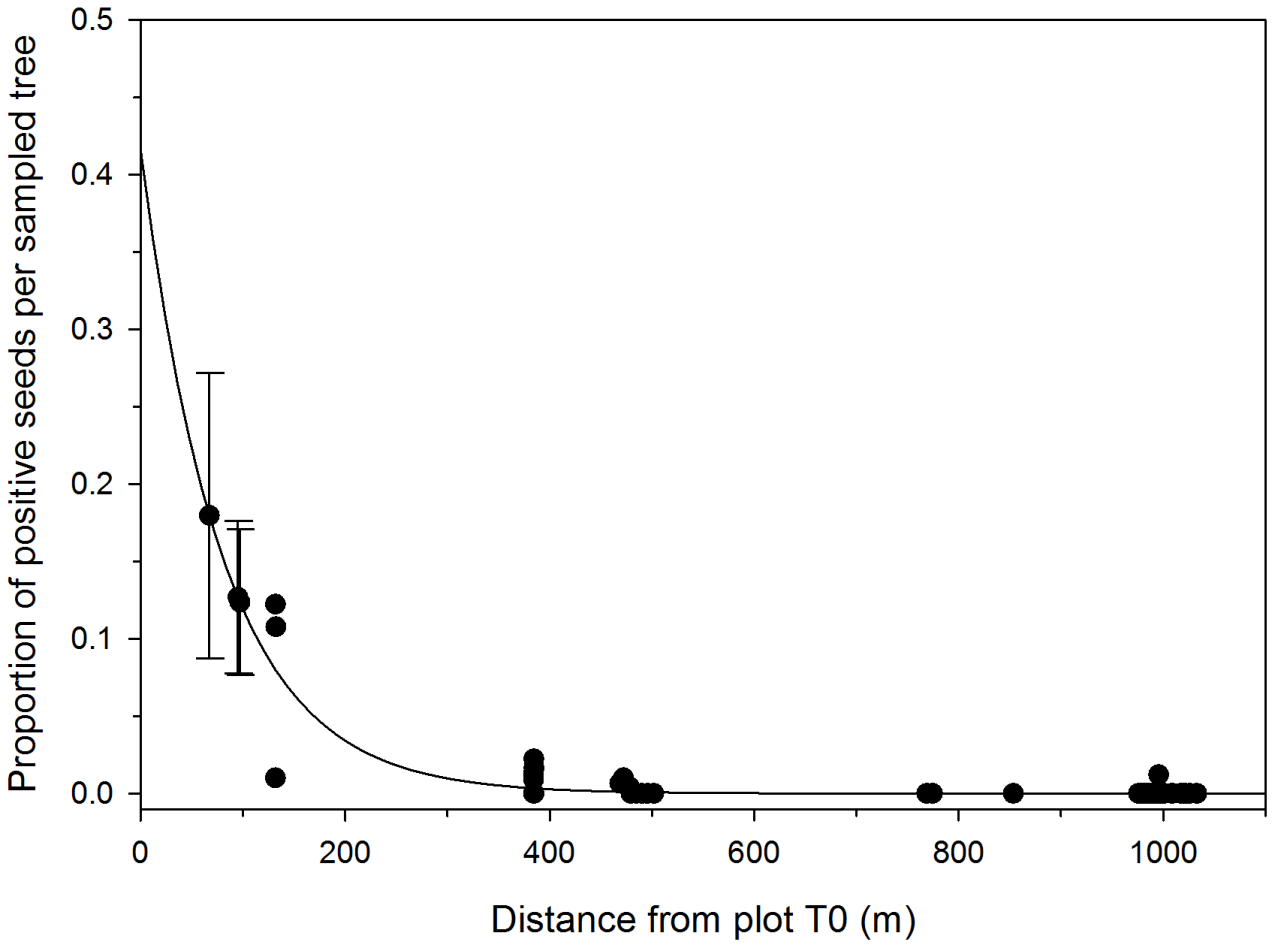
Estimates of the proportion of positive seeds per sampled tree for the 52 sampled tree locations outside of the GE plot (Plot T0) from 2000–2010, based on the fit of the exponential dispersal model to the data. Predictions and 95% prediction intervals are shown for the three tree locations surveyed in plot T0 (distances of 67, 95, and 97 m).

Pollen mediated gene flow outside of plot T0 was detected in 4 of 11 years (2000, 2006, 2007, 2008). Calculations of gene flow are presented as: 1) the percentage of GUS positive embryos over all 12,116 embryos (seeds) collected over the 11-year period of the study (Fig. 2); 2) the percentage of GUS positive embryos over all embryos collected from individual plots (Fig. 2); and 3) the proportion of GUS positive embryos per tree (Fig. 3). In methods 1 and 2 (Fig. 2), gene flow distances indicate the number of meters from the center of the T0 transgene source plot to the center of each sampling plot. Whereas for method 3, distance is the number of meters from the center of the T0 transgene source plot to each individual sampled tree. (Table 2). Regardless of the calculation method used, the results produce the same general view of gene flow in terms of distance from the T0 source block and environmental influences. Based on calculations of gene flow as a percentage of the total number of embryos sampled (12,116) (method 1), plot S1 at a distance from the center of the T0 plot of 132 m produced 0.215% GUS positive embryos; plot M1 at 384 m produced 0.033% GUS positive embryos; plot S2 at 473 m 0.017%; plot M2 at 484 m 0.017%, and at 998 m plot M3 produced 0.025% GUS positive embryos (Fig. 2). No GUS positive embryos were found at the more distant sentinel plots, S3, S4 or S5 (Table 2, Fig. 2). From a total of 30 GUS positive embryos analyzed by PCR from plots S1, S2, and M1, 77% tested positive for receiving pollen from PRSV-CP trees, 17% from PPV-CP trees, 3% from ‘HoneySweet’, and 3% from the vector control.

Based on the distance from the center of T0 to individual trees that produced GUS positive embryos (Table 2; Fig. 3) the proportion of GUS positive seeds per sampled tree decreased over distance from the GE plot (Table 2). For trees in mixed variety plots (M1– M3), on average 0.025% of the embryos tested per tree were positive for the GUS gene. The highest proportions of GUS positive embryos were from plot S1, the closest plot to the GE trees. Over the 11 years surveyed, GUS positive embryos were identified in trees from five of the eight plots. Plot M3, which was the furthest plot from the GE plot (997 m) had one positive tree in one year in which three embryos out of 247 tested from that tree in that year were GUS positive.

### Spatial Pattern of Gene Flow

The probability of bees flying in the area of the sampled trees over the 11 years was uniform (data not shown), and there was no relationship between the amount of GUS positive seeds for each sampled tree and its distance to the bee hives. Therefore, no further analysis on the contribution of managed bees to gene flow was investigated.

The relationship of pollen flow and distance was examined. An exponential dispersal model (y = 0.417*exp(-s/79.94)) was chosen to estimate the proportion of positive seeds per sampled tree (y) based on the trees distance to the GE plot (s) (Fig. 3). The model provided a moderately good fit (R^2^ = 0.67) for all of the sampled tree locations, but there are likely other sources of variation that were not accounted for. The model has a very steep curve at distances less than 400 m from the source and quickly flattens to near zero for further distances (Fig. 3). With this model, only 1% of the seeds per a sampled tree that is 298 m from the source would be expected to be GUS positive. At slightly further distances of 354 and 482 m from the source, 0.5% and 0.1%, respectively, of the seeds per sampled tree should be positive. Non-GE trees that were located inside of plot T0 were not included in the model fit, but these values were extrapolated from the exponential model and predicted to have between 4 and 18% GUS positive seeds (Fig. 3). As stated previously, the observed values at these tree locations were between 4 and 39%.

### Weather Assessment

In general, temperature and wind related weather variables had a positive association with the proportion of GUS positive seeds, and moisture-related variables had a negative association (Table 3). The strongest correlation was for the relationship between rainfall over the bloom period and the proportion of GUS positive seeds (Table 3). This was followed by a created variable that represents the amount of time during the bloom period with warm temperatures and no rainfall. Warmer temperatures coupled with low rainfall are similar to the environmental conditions in which bees prefer to fly [52], [53]. Therefore, an increase in time with these conditions during the bloom period may affect the amount of gene flow. However, there were no significant relationships between any of the weather variables and the average or maximum dispersal distance of GUS positive seeds.

**Table 3:** Correlation coefficient between weather variables and the proportion of trees surveyed [excluding trees in the transgenic plot (T0)] with GUS positive seeds for 7 years (2004–2010) with mean and standard deviation of each weather variable during years where gene flow was observed (2006, 2007, 2008) or was not observed (2004, 2005, 2009, 2010).

Weather variable	Correlation coefficient	Mean and standard deviation during flow years	Mean and standard deviation during non-flow years
Air temperature (C)	0.505	12.671(0.591)	12.662(3.740)
Air temperature >9 C^a^	0.561	0.684(0.039)	0.602(0.178)
Rainfall (mm)	−0.732*	0.015(0.016)	0.021(0.020)
Relative humidity (%)	−0.386	50.908(1.656)	52.047(12.807)
Air temperature >9 C and no rainfall^b^	0.694*	0.673(0.046)	0.596(0.174)
Wind speed (m/s)	0.589	2.621(0.600)	2.244(0.418)
Hourly evaporation (mm)	0.549	0.099(0.054)	0.078(0.023)
Total PAR (µmol/m^2^/s)	0.063	49.660(4.502)	48.825(15.338)

* *P*<0.10.

a Proportion of 10-minute increments during the bloom period each year where air temperature was greater than 9 C.

b Proportion of 10-minute increments during the bloom period each year when it was not raining and air temperature was greater than 9 C.

### Native Pollinators

During all years of the study, there was a mix of native pollinators and honeybees from managed hives acting to pollinate the plum trees in this study. The early onset of warm temperatures in 2012 prior to the arrival of the managed bee hives allowed for the sampling of native pollinators although freezing temperatures following the warm period eliminated plum fruit production, therefore, the efficiency and gene flow provided by these native pollinators could not be evaluated. The identification of putative native and naturalized pollinating insects and their abundance is shown in Table 4.

**Table 4:** Native and naturalized insect pollinators in GE plum trees (Plot T0) and non-GE trees (Plot M3).

Pollinator	% total capture	% capture Plot T0	% capture Plot M3
*Andrena miserabilis*	26	24	2
*Andrena imitatrix*	23	14	9
flies	17	4	13
*Colletes inaequalis*	7	4	3
Winged ants	5	5	0
*Andrena carlini*	3	2	1
Other species*	19	8	11

Total capture was 193 (120 in plot T0 and 73 in plot M3).

* Andrena rugosa, cressonii, nasonii, dunning, bisalicis, imbricate; Lasioglossum gotham; Osmia taurus; Ceratina calcarata; Halictus confuses; Augochlora pura; Polistes dorsalis.

### Seed Mediated Gene Flow

We scouted 12.1 ha of naturalized, forested for flowering *P. domestica* seedling trees (Fig. 2) and none were found. Juvenile non-flowering (3–5 year old) seedlings could have been overlooked in this survey. While any observations of non-flowering seedlings would not be ignored, their presence might not have been apparent.

It is important to consider that any plum seedlings that may have developed due to seed-based gene flow over the life of all plum plantings on the property planted since 1979 would have been observed. No *P. domestica* trees were found in cultivated plantings of other tree fruits on the property and would not be expected based on the use of herbicides and other cultivation methods that would eliminate any volunteer plants.

## Discussion

Transgene flow out of the GE plum block only occurred in four of the 11 years of the study. When it did occur, we provided three calculations of gene flow based on the total number of seeds collected over the life of the study (Table 2, Fig. 2), based on the total number of seeds collected over the life of the study on a per plot basis (Table 2, Fig. 2), and based on the proportion of GUS positive embryos collected from individual trees over the life of the study (Table 2, Fig. 3). While each calculation provides the same overall view of gene flow over the course of the 11-year study, a particular method may be of interest to growers and regulatory officials concerned with issues of co-existence.

During the first 8 years of the study, 67 GE source trees in plot T0 (Fig. 1) contributed to gene flow. Recipient non-GE trees from 2000–2005 were located in plots M1, M2, and M3 (Fig. 2). During this period only two GUS positive seeds were identified from 4,416 seeds analyzed (0.045%). From 2005–2010, 19 GE trees were sources of transgene flow and recipient trees were in plots M1, M2, M3 and in sentinel plots S1–S5 (Fig. 2). Sentinel plots had been planted in 2003 and began flowering and producing a small amount of fruit in 2005. From 2006 through 2010, 35 GUS positive seeds were identified from 7,700 seeds sampled (0.45%). Of the 35 GUS positive seeds identified during this period, 24 were collected from two sentinel trees in plot S1 in 2007. This was the plot closest to T0. These samples, collected in a single year accounted for 65% of the total gene flow for the entire 11-year study. In addition to the close proximity of plot S1 to T0, the 2007 spring season had the earliest and longest span of temperatures >9°C without rainfall of the recorded years. These early warm temperatures may have concentrated the flowering period into a short duration, and dry warm weather conditions would favor insect-mediated cross-pollination [52]. In 2007 the highest rate of gene flow to monitoring plots (although still quite low) was also recorded, four GUS positive seeds in plot M1 and one in S2.

The long-term nature of the study provided for a variety of conditions that may be considered typical for plum growing areas. These include weather-related effects on the timing of flowering that would influence the potential for overlap of bloom between different varieties, and influences on pollinating insect activity. Spring in some years of the study was characterized by days of high temperature and little rainfall favoring a concentration of bloom and relatively high levels of pollinating insect activity, increasing the potential for cross pollination and gene flow (e.g. 2007). Some years were characterized by slow warming with warm days intermingled with cool days that maximized the differences in bloom time between early and later blooming cultivars and reduced the chances of cross-pollination. Rainfall during the time of bloom in some years reduced the activity of most insect pollinators, reducing gene flow. These climatic variations are typical in virtually all *P. domestica* growing regions. Cultivars naturally varying in bloom time and with different levels of sexual compatibility would also be typical of many plum-growing regions. The conditions of our study in particular resembled those typical of small grower plots separated by sexually non-compatible species such as peach, apple and pear, and by small patches of wood- and grass-land. The presence of a double row of peach trees around the T0 plot required by APHIS differed from a typical commercial planting of *P. domestica.* Whether this double row significantly influenced gene flow could not be assessed in this study. Small-holdings of mixed plantings of various species of fruit trees are not uncommon in many plum growing areas and perhaps not so unlike the presence of peach trees surrounding the T0 plot.

The conditions of our study favored gene flow by including *A. melifera* bee hives which, along with natural pollinators such as *Osmia* spp. that are known to pollinate Rosaceous species [17], appears to have provided a condition of saturation of pollinators [53]. The higher levels of native or naturalized pollinators found in plot T0 when compared with those recorded in plot M3 (Table 4) may have been due to the fact that T0 is surrounded on three sides by naturalized woodland areas which would have provided habitat for these species [55]–[56].

Most of the trees evaluated for gene flow were confirmed as sexually compatible with ‘HoneySweet’ GE plum (ex. ‘Bluebyrd’, ‘Italian Prune’, ‘Seneca’, ‘Jojo’, ‘Cacanska lepotica’, ‘Pozegaca’, ‘President’, ‘Reine Claude’). ‘HoneySweet’ was related to most other GE plums in the GE block, having the same female parent; therefore the probability of cross compatibility between these ‘HoneySweet’ sibling trees and the sampled trees was high. The highest level of gene flow was recorded from the PRSV-CP trees that were not related to ‘HoneySweet’. Higher levels of gene flow from these trees may be explained at least in part by a higher level of cross-compatibility with recipient trees but was most likely due to their location in the T0 plot, being in the closest proximity to the sentinel planting (S1) from where most of the gene flow to non-GE plum trees was recorded (Table 2, Fig. 2). PCR analyses of gene flow to plot S1 indicated that of 24 embryos tested, 20 were positive for receiving pollen from PRSV-CP trees. These trees were in closest proximity to trees in plot S1 at a distance of 28–42 m.

Over the 11-year period, gene flow was recorded only to trees of ‘Seneca’, ‘Italian Prune’, ‘Bluebyrd’, and plum seedlings in plot M1.

It was interesting to note that no feral plum trees were observed in the naturalized areas of vegetation on the research station grounds, even in areas adjacent to plum blocks (Fig. 2). Considering that plum plots were planted in 1979, 1992, 1995, 1996, 1997, and 2005, the absence of feral trees suggests that seed-mediated gene flow in *P. domestica* under the conditions described in this study is extremely low, specifically, non-detectable. Seed dispersal in some Prunus species is aided by avian frugivores [57] and although a number of avian species could be found visiting and even nesting in trees of the sampled plots, including T0, they did not appear to influence seed distribution, perhaps due to the relatively large size and hardness of *P. domestica* seeds.

Gene flow in plum is most appropriately compared with gene flow in other temperate and subtropical entomophilous tree fruit species. Consistently, gene flow in these species is low (<1–3%) and inversely related to distance. In almond, gene flow is has been shown to take place mostly with neighboring trees to the extent that the half of the recipient tree facing away from the pollen source tree received significantly less gene flow [58]. In apple, gene flow was recorded mostly between neighboring trees with maximum distances for gene flow recorded at 300 m with a mean of 60 m [59]. Soejima [60] found gene flow in apple at distances of 60–150 m. In a two-year study of apple gene flow, Reim et al. [61] reported that 91% of gene flow occurred at less than 60 m from donor trees. Tyson et al. [21] reported apple gene flow over two years to be limited to less than 146 m with a precipitous decline beyond 30 m. They found that the use of a buffer row significantly reduced gene flow. PMTF studied for seven years in citrus showed a low level of gene flow (0.17–2.86%) and a strong relationship with distance from the pollen donor source [20]. Based on their work a border of non-GE trees was recommended to provide pollinators with a competing pollen source.

Over a period of 11 years we demonstrate that gene flow from genetically engineered plums is low, only detected in four of 11 years. When gene flow was detected, it was related to the distance from the source block and related to environmental conditions known to favor gene flow. These conditions, warm temperatures and little or no rain are not consistently encountered in the study area or in most *P. domestica* plum growing areas. The placement of managed honeybee hives throughout the study area insured sufficient pollinator activity and was naturally supplemented by native pollinators. Spatial modeling of gene flow revealed a dramatic reduction at distances of less than 400 m. Over the 11 years of sampling, gene flow reached only 0.31% (37/12,116 seeds analyzed). When this low rate of pollen-mediated gene flow is combined with the lack of seed movement into natural or naturalized habitats, it is clear that the overall potential for gene flow in this species is extremely low. Further, when considering gene flow, unlike other species that may produce a large number of seeds per a single floral visit (e.g. *Fragaria*, *Malus*) each *P. domestica* flower produces only one seed adding to the limitation of pollen-mediated gene flow. Finally, in terms of the movement of transgenes from the orchard into the market, in the case of plum and most tree fruits, gene flow is to the seed, which is neither consumed, nor typically planted, as grafting is the normal route of tree fruit propagation. These results, low pollen-mediated gene flow, undetectable seed-mediated gene flow, low seed production per pollinator visit, and lack of utility of the seed, together demonstrate the high potential for coexistence of GE and non-GE plum trees.
